# Supplementary material for: Genome-Wide Analysis of the Cis-Prenyltransferase (CPT) Gene Family in Taraxacum kok-saghyz Provides Insights into Its Expression Patterns in Response to Hormonal Treatments
Source: Plants (Basel). 2025 Jan 27;14(3):386. doi: 10.3390/plants14030386 (PMC11820359; doi:10.3390/plants14030386)
Supplement: Supplementary file 1 [file plants-14-00386-s001.zip › Figure S1 TkCPT and TkCPTL gene family protein tertiary structure.pdf]

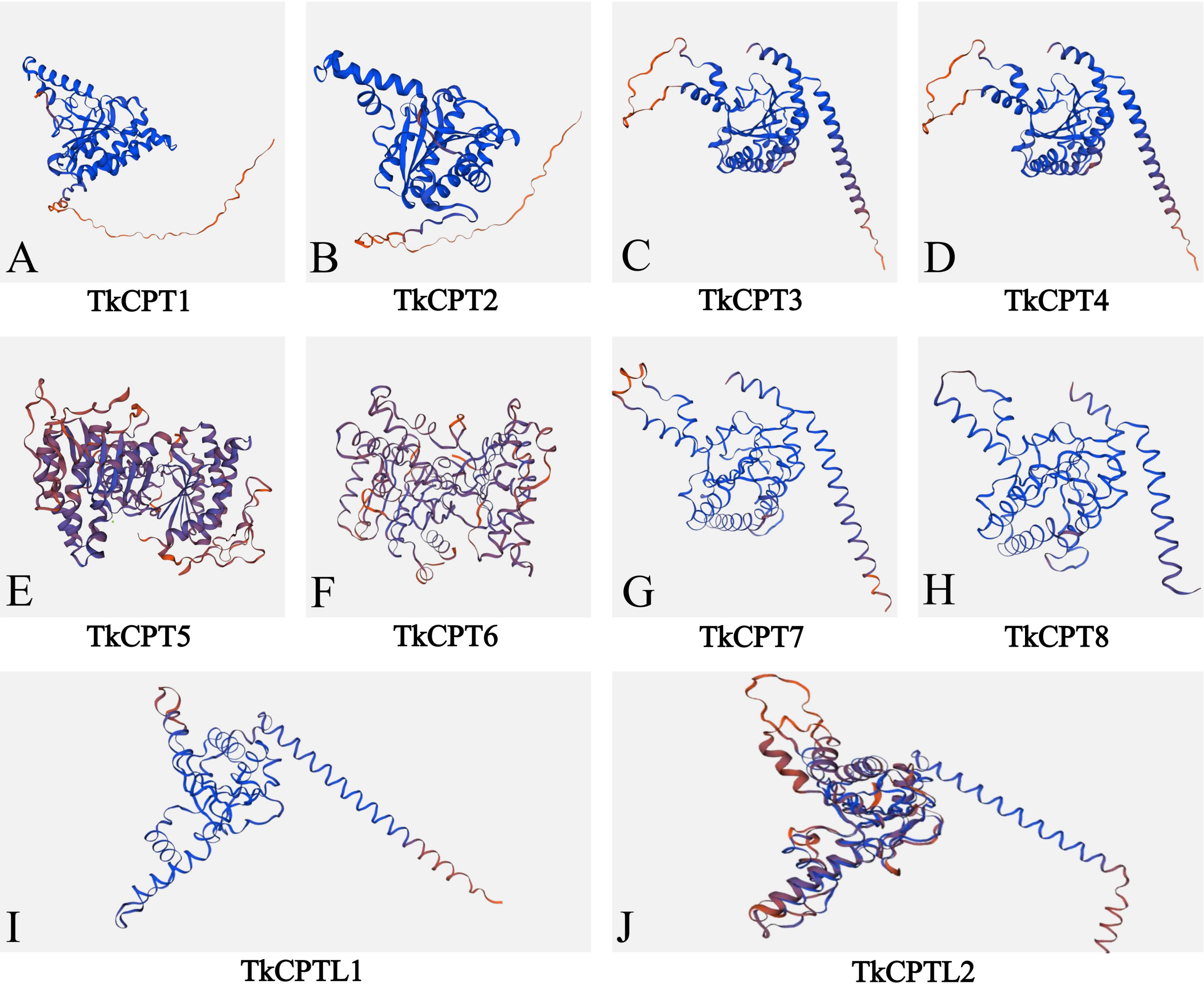


Figure. S1. Proposed TkCPT/TkCPTL 3D structural model. The letters in the upper right corner represent the order of all 3D structures. TkCPT1 and TkCPT2 are relatively similar, TkCPT3 and TkCPT4 are relatively similar, TkCPT5 and TkCPT6 are relatively similar, and TkCPTL1 and TkCPTL2 are relatively similar, which may be related to the function of their forms.
