# Supplementary material for: Genome-Wide Analysis of the Cis-Prenyltransferase (CPT) Gene Family in Taraxacum kok-saghyz Provides Insights into Its Expression Patterns in Response to Hormonal Treatments
Source: Plants (Basel). 2025 Jan 27;14(3):386. doi: 10.3390/plants14030386 (PMC11820359; doi:10.3390/plants14030386)
Supplement: Supplementary file 1 [file plants-14-00386-s001.zip › Figure S2 Prediction of transmembrane helices of TkCPT and TkCPTL.pdf]

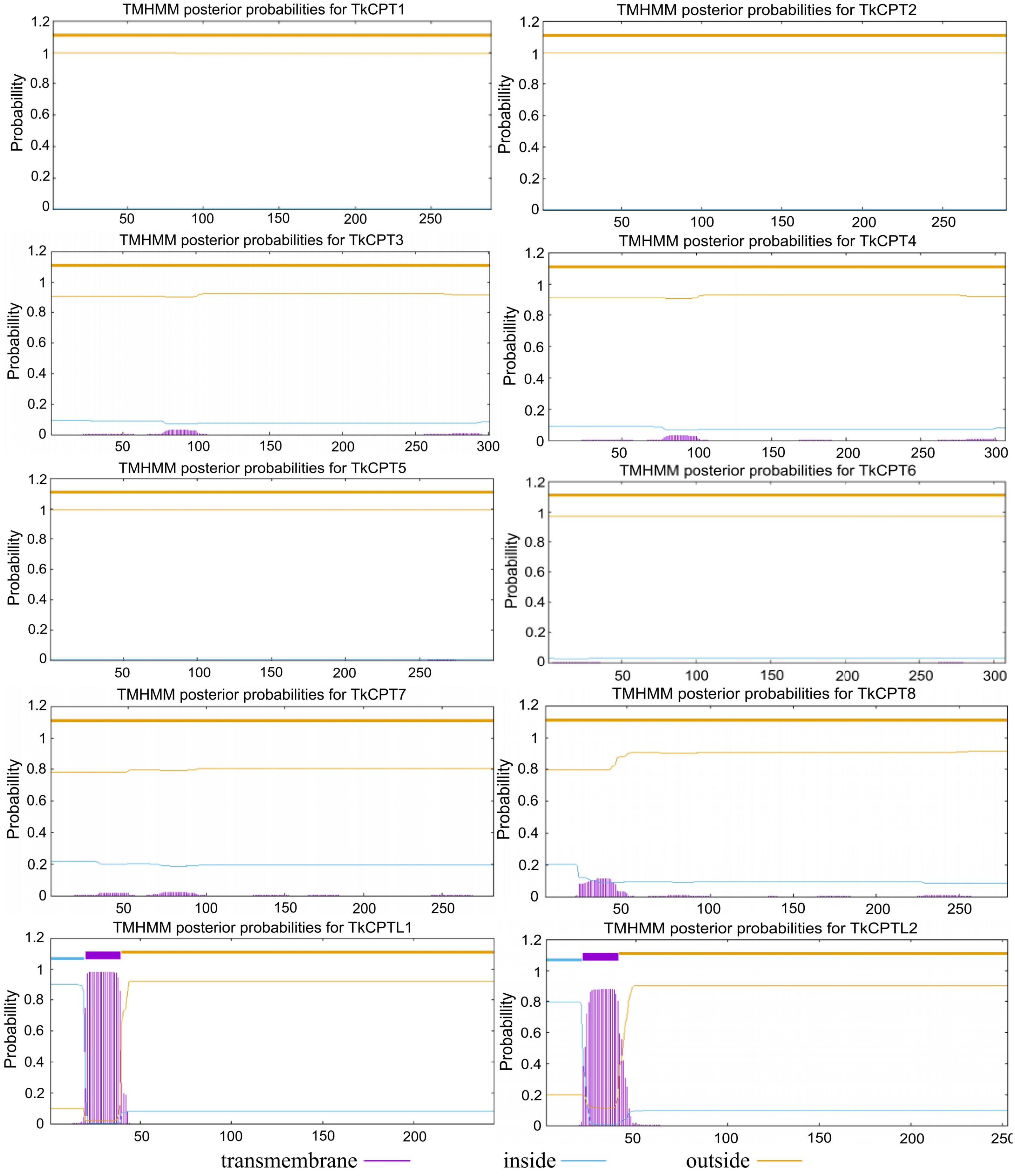

Figure. S2.The prediction of CPT/CPTL transmembrane structure in *Taraxacum kok-saghyz* is represented by three colored lines.The yellow line represents the outer region, the blue line represents the inner region, and the purple line represents the transmembrane domain.
