# Supplementary material for: Genome-Wide Analysis of the Cis-Prenyltransferase (CPT) Gene Family in Taraxacum kok-saghyz Provides Insights into Its Expression Patterns in Response to Hormonal Treatments
Source: Plants (Basel). 2025 Jan 27;14(3):386. doi: 10.3390/plants14030386 (PMC11820359; doi:10.3390/plants14030386)
Supplement: Supplementary file 1 [file plants-14-00386-s001.zip › Figure S3 Promoter cis-elements analysis of TkCPT and TkCPTL .pdf]

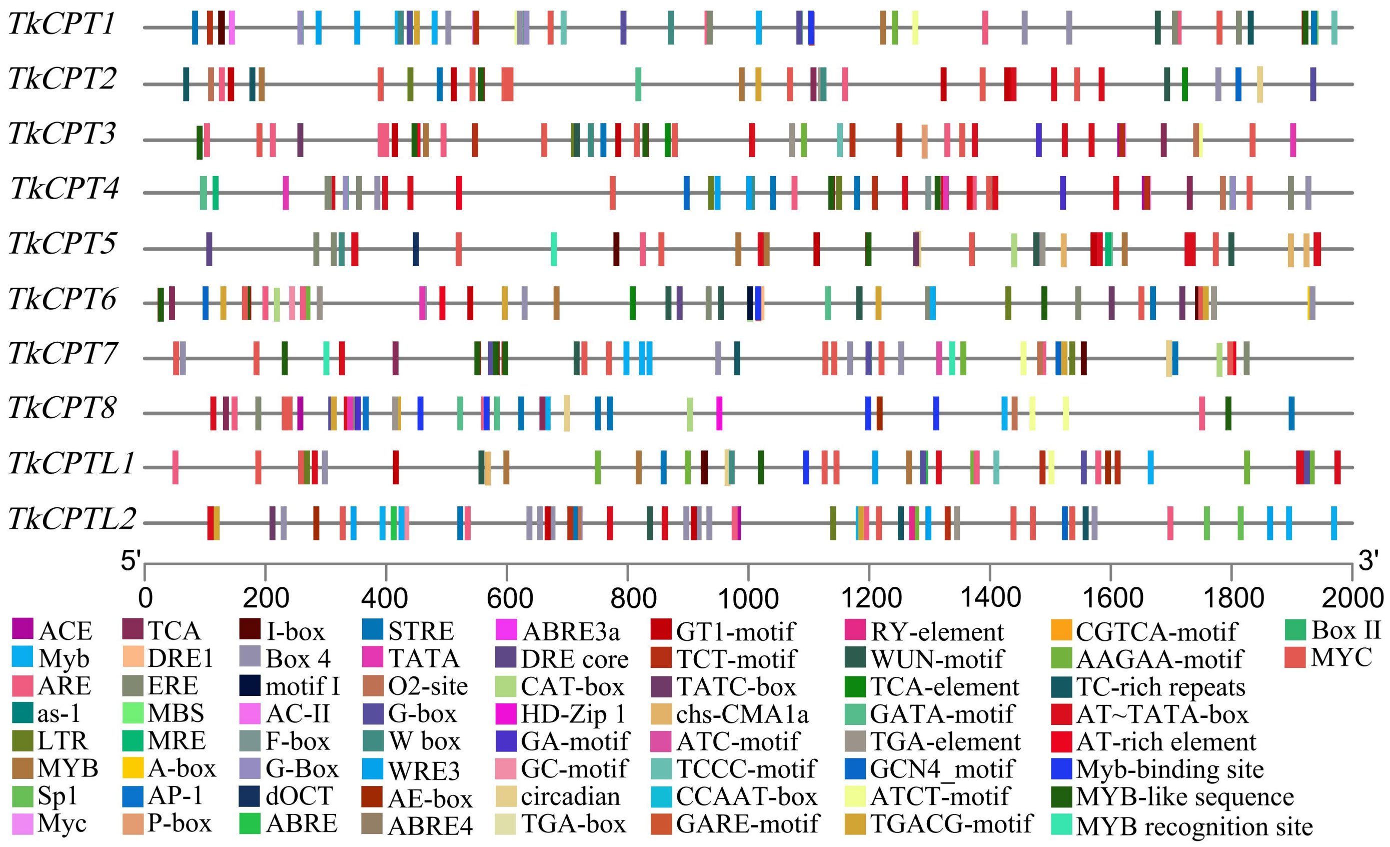

Figure. S3. Promoter cis-elements analysis of TkCPTs. The different types of cis-elements are represented by different shapes colors.
