# Supplementary material for: Genome-Wide Analysis of the Cis-Prenyltransferase (CPT) Gene Family in Taraxacum kok-saghyz Provides Insights into Its Expression Patterns in Response to Hormonal Treatments
Source: Plants (Basel). 2025 Jan 27;14(3):386. doi: 10.3390/plants14030386 (PMC11820359; doi:10.3390/plants14030386)
Supplement: Supplementary file 1 [file plants-14-00386-s001.zip › Figure S4 Multiple alignments of TkCPT and TkCPTL amino acid sequences..pdf]

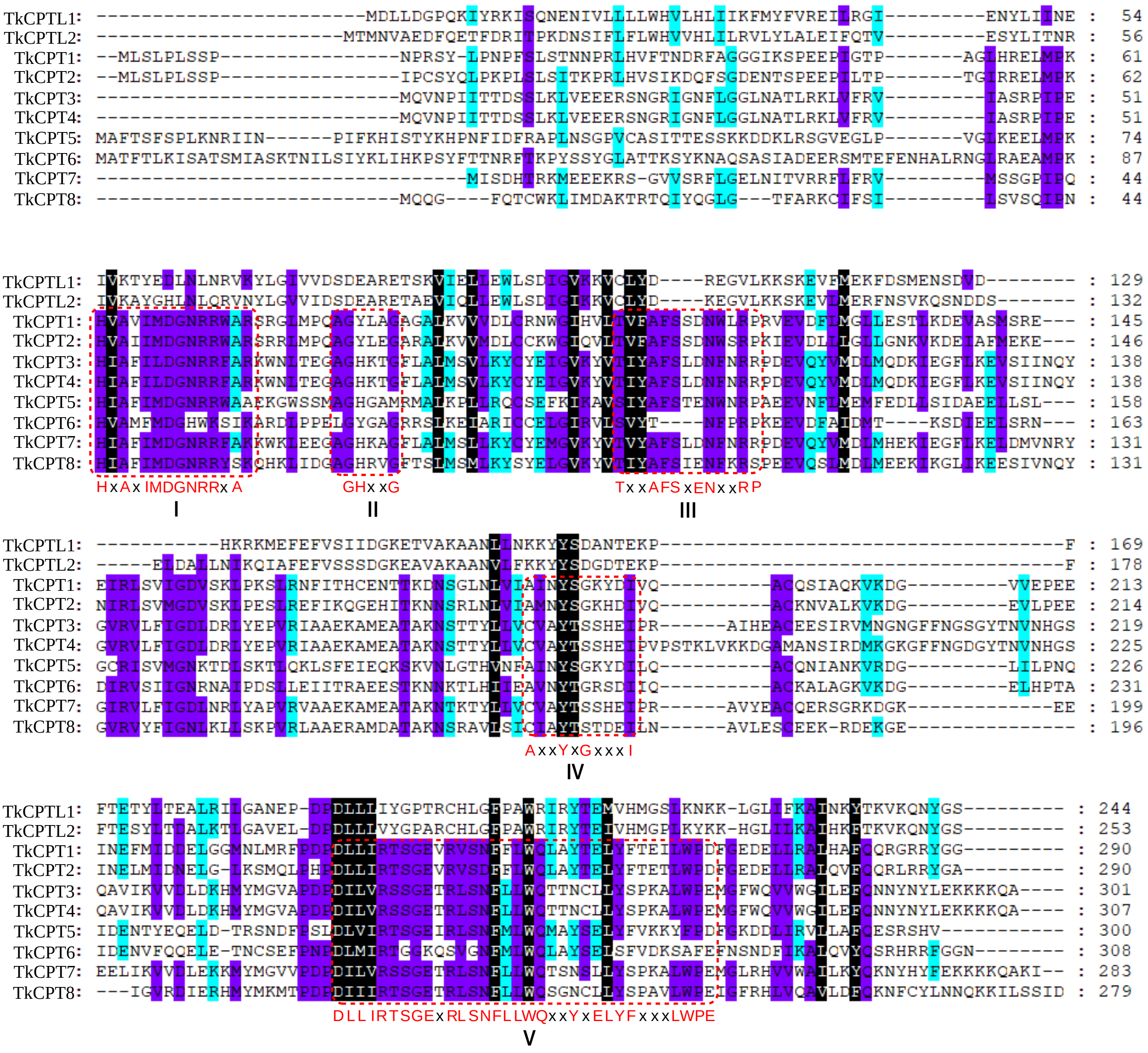

Figure S4. Multiple alignment of amino acid sequences in the TkCPT/CPTLs family. Different colors used to highlight amino acid residues indicate the degree of amino acid identity. The black line represents the position of a completely conserved motif. Pink represents a 70% conservative rate, while blue represents a 60% conservative rate. The structural domains enclosed by red dashed lines represent the five characteristic conservative regions (I-V) of TkCPT.
