# Supplementary material for: Genome-Wide Analysis of the Cis-Prenyltransferase (CPT) Gene Family in Taraxacum kok-saghyz Provides Insights into Its Expression Patterns in Response to Hormonal Treatments
Source: Plants (Basel). 2025 Jan 27;14(3):386. doi: 10.3390/plants14030386 (PMC11820359; doi:10.3390/plants14030386)
Supplement: Supplementary file 1 [file plants-14-00386-s001.zip › Figure S5 TkCPT and TkCPTL conserved core consensus sequence.pdf]

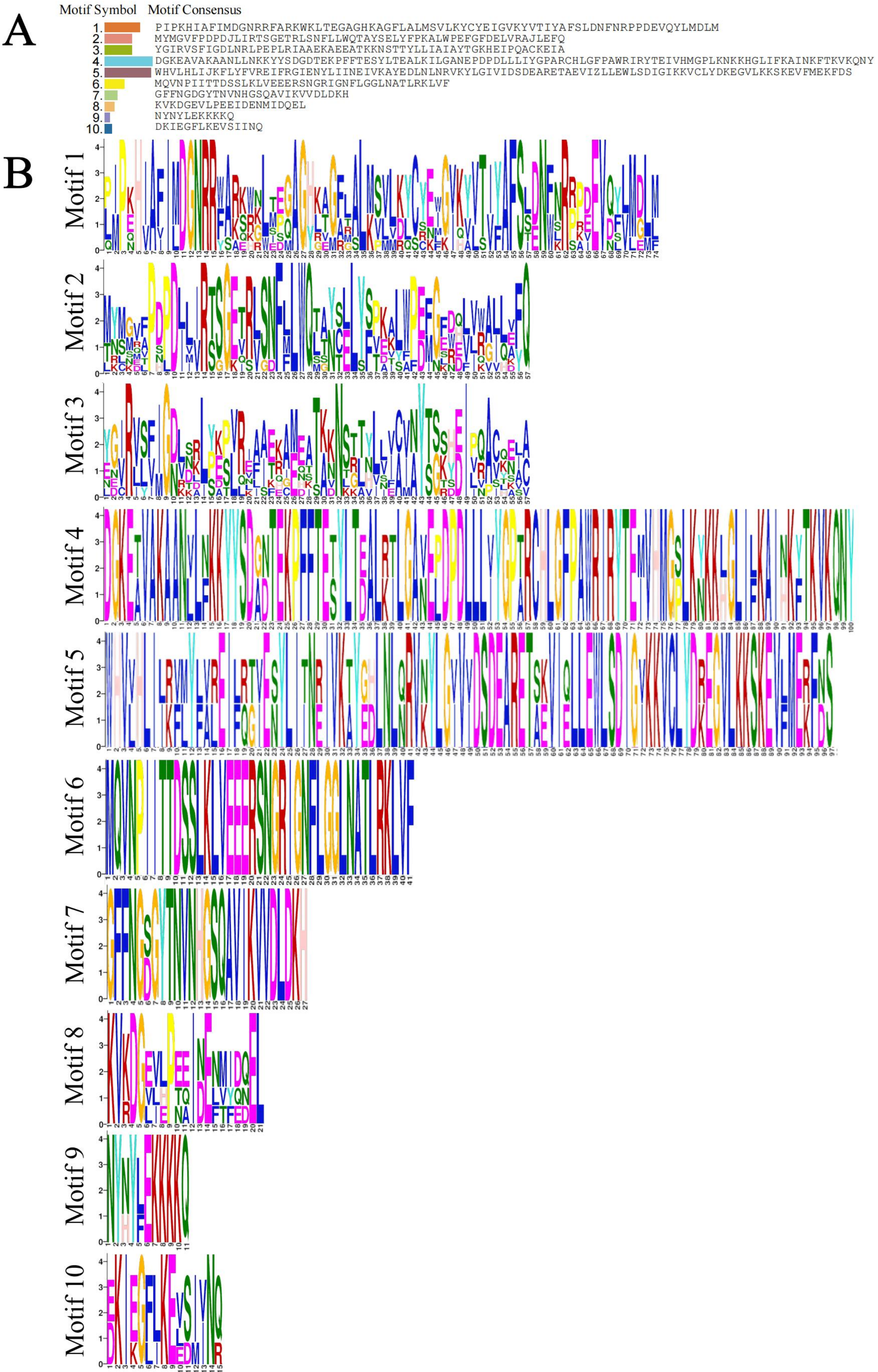


Figure. S5. (A) Motif symbol and motif consensus of TkCPT/CPTLs. On the left are different motif  symbol, and on the right are all motif protein sequences of TkCPT/ CPTLs. (B) Logo of motif for conserved consensus sequence of TkCPT/CPTLs. The overall height in eachstack indicates sequence conservation at that position; the height of each residue letter indicates therelative frequency of the corresponding residue (color figure online). The scale at the bottom is usedto compare the lengths of different proteins.
